# Supplementary material for: FORMATION OF MULTINUCLEATED OSTEOCLASTS DEPENDS ON AN OXIDIZED SPECIES OF CELL SURFACE ASSOCIATED LA PROTEIN
Source: bioRxiv. 2024 Jul 3:2024.05.02.592254. Originally published 2024 May 5. Preprint. [Version 2] doi: 10.1101/2024.05.02.592254 (PMC11188106; doi:10.1101/2024.05.02.592254)
Supplement: Supplement 1 [file NIHPP2024.05.02.592254v2-supplement-1.pdf]

bioRxiv preprint doi: https://doi.org/10.1101/2024.05.02.592254; this version posted July 3, 2024. The copyright holder for this preprint (which was not certified by peer review) is the author/funder. This article is a US Government work. It is not subject to copyright under 17 USC 105 and is also made available for use under a CC0 license.

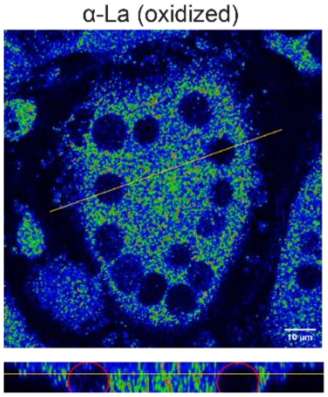

Supplementary Figure 1. Oxidized La is found in the cytosol. 3D stack image depicting the cytosolic localization of oxidized LA. Top - XY-slice slightly below the equatorial plane of a permeabilized multinucleated osteoclast (3 days post-RANKL application) stained with an α-La antibody that recognizes oxidized La. 3D stack of this representative cell was acquired with 0.22μm step interval. Bottom - Z-slice through the orange line on the top panel. Red ellipses show the approximate outlines of two nuclei in the slice, and the orange line shows the location of the XY-slice shown on top.

bioRxiv preprint doi: https://doi.org/10.1101/2024.05.02.592254; this version posted July 3, 2024. The copyright holder for this preprint (which was not certified by peer review) is the author/funder. This article is a US Government work. It is not subject to copyright under 17 USC 105 and is also made available for use under a CC0 license.

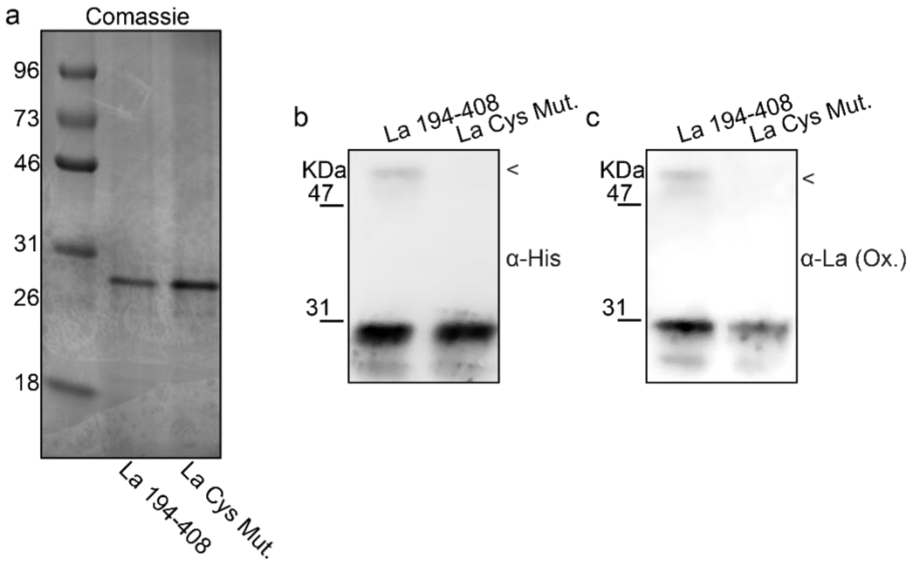

Supplementary Figure 4: La C-terminal half and cysteine mutant purification. (a) A gray-scale image of La 194-408 or cysteine mutant La 194-408 separated via polyacrylamide gel electrophoresis and visualized using Coomassie staining. Representative Western Blots depicting La C-terminal half and cysteine mutant recognized by α-6xhis **(b)** or α-La (ox.) α-6xhis **(c)**. < Denotes the migration of La 194-408 as a dimer.

bioRxiv preprint doi: https://doi.org/10.1101/2024.05.02.592254; this version posted July 3, 2024. The copyright holder for this preprint (which was not certified by peer review) is the author/funder. This article is a US Government work. It is not subject to copyright under 17 USC 105 and is also made available for use under a CC0 license.

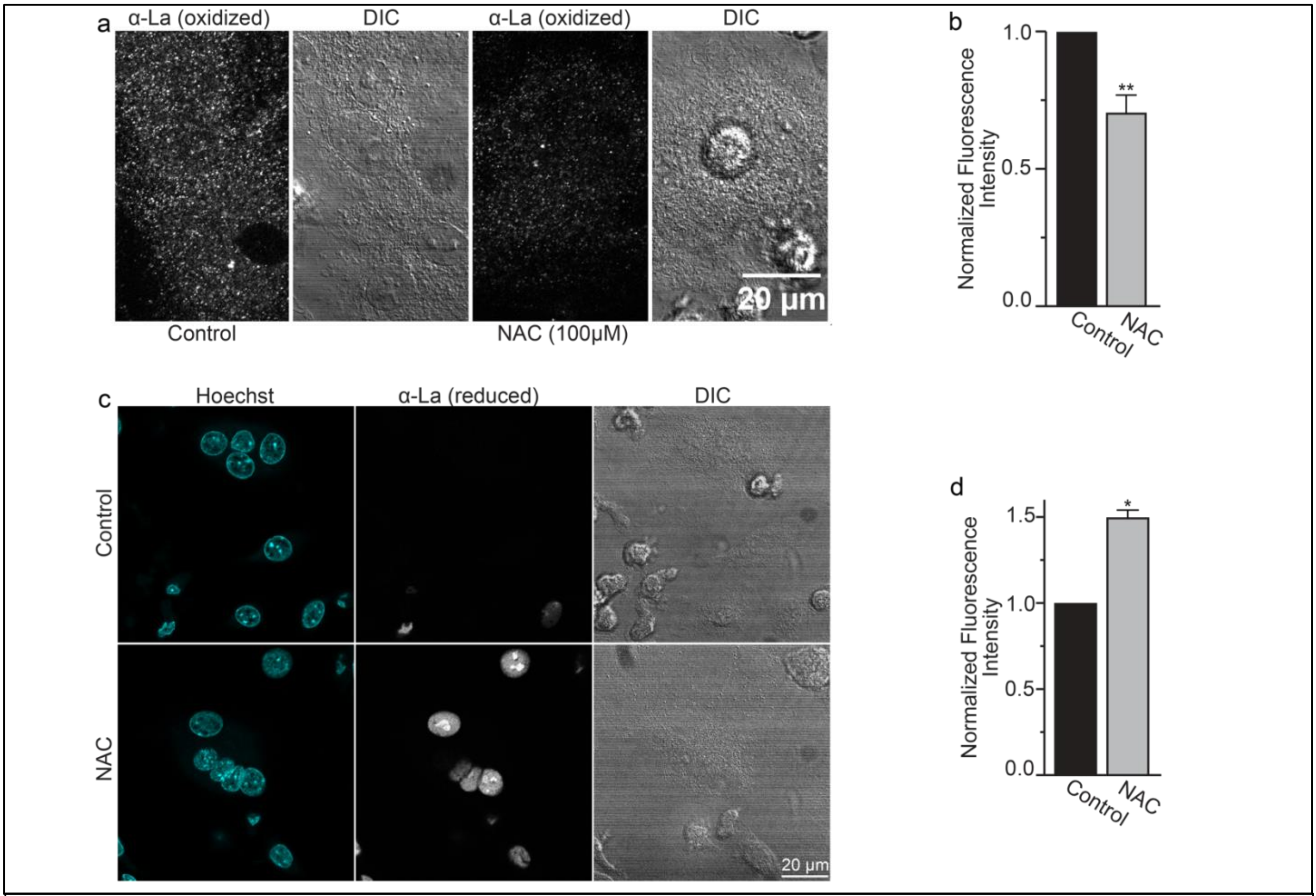

Supplemental Figure 5. NAC inhibits redox shift from reduced to oxidized species of intracellular La. **(a)** Fluorescence microscopy and DIC images of permeabilized osteoclasts treated or not treated (control) with 100 µm (1h) NAC and stained with an α-La antibody that recognizes oxidized La at 3 days post RANKL application. **(b)** Quantification of **a**. (n=5) (p=0.009). **(c)** Fluorescence microscopy and DIC images of permeabilized osteoclasts treated or not treated (control) with 100 µm (1h) NAC and stained with an α-La antibody that recognizes reduced La at 3 days post RANKL application. **(d)** Quantification of **c**. (n=2) (p=0.04). Statistical significance evaluated via paired t-test.
